# An Evaluation of the Cytotoxic and Genotoxic Effects of the Marine Toxin C17-SAMT in Human TK6 and HepaRG Cell Lines

**DOI:** 10.3390/ijms24097805

**Published:** 2023-04-25

**Authors:** Zeineb Marzougui, Ludovic Le Hegarat, Kevin Hogeveen, Sylvie Huet, Riadh Kharrat, Riadh Marrouchi, Valérie Fessard

**Affiliations:** 1 Laboratoire des Venins et Biomolécules Thérapeutiques, Institut Pasteur de Tunis, Université Tunis El Manar, 13 Place Pasteur, B.P. 74, Tunis-Belvédère 1002, Tunisia; zeineb.marzougui@inat.u-carthage.tn (Z.M.); riadh.kharrat@pasteur.tn (R.K.); 2 Institut National Agronomique de Tunisie, Université de Carthage, Tunis 1082, Tunisia; 3 Unité de Toxicologie des Contaminants, Agence Nationale de Sécurité Sanitaire (ANSES), 10 B rue Claude Bourgelat, 35306 Fougères, France; ludovic.lehegarat@anses.fr (L.L.H.); kevin.hogeveen@anses.fr (K.H.); sylvie.huet@anses.fr (S.H.); valerie.fessard@anses.fr (V.F.)

**Keywords:** marine toxins, C17-SAMT, in vitro, micronucleus assay, oxidative stress, mitochondrial dysfunction, γH2AX, pH3 phospho S10

## Abstract

This study investigates the genotoxicity and cytotoxicity of C17-sphinganine analog mycotoxin (C17-SAMT) using in vitro assays. C17-SAMT was previously identified as the cause of unusual toxicity in cultured mussels from the Bizerte Lagoon in northern Tunisia. While a previous in vivo genotoxicity study was inconclusive, in vitro results demonstrated that C17-SAMT induced an increase in micronucleus formation in human lymphoblastoid TK6 cells at concentrations of 0.87 µM and 1.74 µM. In addition, multiparametric cytotoxicity assays were performed in the human hepatoma HepaRG cell line, which showed that C17-SAMT induced mitochondrial dysfunction, decreased cellular ATP levels, and altered the expression of various proteins, including superoxide dismutase SOD2, heme oxygenase HO-1, and NF-κB. These results suggest that C17-SAMT is mutagenic in vitro and can induce mitochondrial dysfunction in HepaRG cells. However, the exact mode of action of this toxin requires further investigation. Overall, this study highlights the potential toxicity of C17-SAMT and the need for further research to better understand its effects.

## 1. Introduction

The total number of fungal species on earth is not clear, but estimated values range between 1.5 and 5 million [1]. In adequate environmental conditions, some fungi produce secondary metabolites and mycotoxins [2] that can be found throughout the food chain [3]. So far, around 400 mycotoxins have been identified, originating primarily from filamentous fungi including *Alternaria*, *Aspergillus*, *Fusarium*, *Penicillium*, etc., [4,5]. Transported along the food chain, mycotoxins cause irreversible damage in humans and animals. Indeed, mycotoxins have been shown to affect multiple organs and tissues including the liver, kidney, and immune and nervous systems [4,6]. The severity of health effects depends on multiple factors following exposure to the toxin, including environmental circumstances, mixture with other contaminants, and individual health problems, such as immunodeficiencies [4]. The substantial risk to human and animal health has, therefore, attracted international concern [7].

While data on fungi and mycotoxins are rather well-established in terrestrial ecosystems, much less is known about fungi and mycotoxins present in the marine environment [1]. Fungi have been found and identified in every compartment of the marine ecosystem and at different depth levels [8,9,10,11,12,13]. In fact, over 1000 fungal species have been exclusively identified in marine ecosystems, with the first record appearing in the 19th century [1]. However, data regarding the toxins produced by marine fungi and their potential toxicity are scarce. Nevertheless, available studies have shown that farmed bivalve mollusks can accumulate mycotoxins, such as gliotoxin produced by *Aspergillus fumigatus* that was found in blue mussels (*Mytulis edulis*) [14]. In the same context, metabolites originating from *Trichoderma* sp. have contaminated sediments and mussels in shellfish farming areas [15]. Recently, aflatoxins (AFs), ochratoxins (OTAs), deoxynvalenol (DON), and zearalenone (ZEN) [16], well-known for their contamination of terrestrial ecosystems and for their hazardous effects to humans and animals, have been also detected in seafood.

In 2013, Marrouchi et al. [17] identified a novel marine mycotoxin that was associated with recurrent and atypical toxicity events in farmed mussels (*M*. *galloprovincialis*) from the Bizerte lagoon in North Tunisia. Preliminary studies with mussel extracts reported severe toxicity symptoms including jumping, dyspnea, flaccid paralysis, and death within a few minutes in the mouse bioassay. To identify the compound responsible for the observed toxicity, a bio-guided chromatographic separation followed by mass spectrometry detection was performed. These analyses confirmed the presence of a 17-carbon short-chain analog to sphinganine, the C17-Sphinganine Analog MycoToxin (C17-SAMT), with a molecular mass of 287.289 Da. Following treatment of mice with the purified toxin through intracerebroventricular, intraperitoneal, and oral routes, LD50 values of 150, 750, and 900 µg/kg b.w were observed, respectively [17]. Recently, an in vivo genotoxicity study showed that C17-SAMT induced equivocal results of DNA damage in the liver with the comet assay, whereas the bone marrow micronucleus assay was negative but without evidence of bone marrow exposure [18].

In order to clarify the genotoxic potential of C17-SAMT, we performed a micronucleus assay on human lymphoblastoid TK6 cells, as recommended by the OCDE guideline No. 487 [19]. The liver is a target of xenobiotic toxicity, and is the principal site for their metabolism [20,21]. The HepaRG cell line represents an interesting metabolically competent in vitro model to investigate the hepatotoxicity of xenobiotic compounds [22]. Differentiated HepaRG cells show similar characteristics to primary human hepatocytes in terms of liver functionality, including the expression of transporters and the inducible expression of metabolic enzymes. To investigate its mechanism of action, we also assessed several toxicity endpoints including γH2AX, pH3 phospho S10, phospho ATM S1981 levels, and mitochondrial membrane potential in the human hepatoma HepaRG cell line. Additionally, we evaluated oxidative stress and pro-inflammatory response.

## 2. Results

### 2.1. In Vitro Micronucleus Test in TK6 Cells

**Figure 1:**
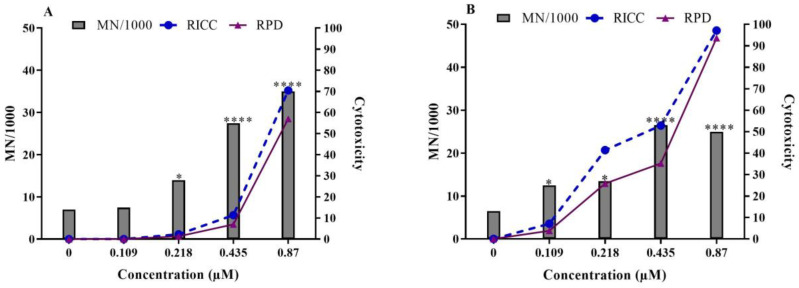
Micronucleus test with C17-SAMT in TK6 cells following a 24 h treatment ((**A**) exp. 1, and (**B**) exp. 2). Graphs represent the frequency of micronucleated TK6 cells. The cytotoxicity is indicated by RICC and relative population doubling (RPD). * *p* < 0.05 and **** *p* < 0.0001.

Following a 24 h exposure, C17-SAMT significantly increased MN formation in TK6 cells. As presented in Figure 1, concentration-dependent increases in MN frequency were observed in both experiments at concentrations from 0.218 µM, with MN frequency reaching 35‰ cells at the highest concentration of C17-SAMT tested. MN induction was observed for at least one non-cytotoxic concentration of C17-SAMT (less than a 60% relative increase in cell count RICC). The positive control MMS (10 µM) increased the frequency of micronucleated cells in the two experiments (39.42 and 43.96‰, respectively).

### 2.2. Multiparametric Cytotoxicity Assays in HepaRG Cells

#### 2.2.1. DNA Damage

- γ-H2AX and phospho-H3

**Figure 2:**
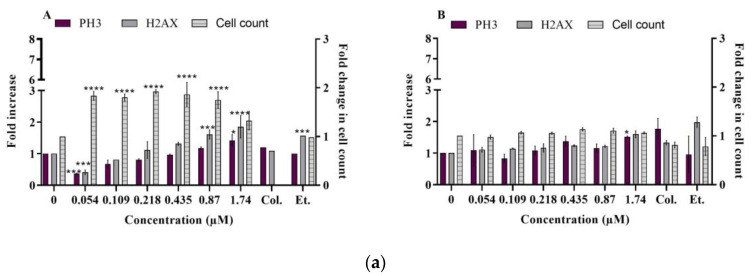

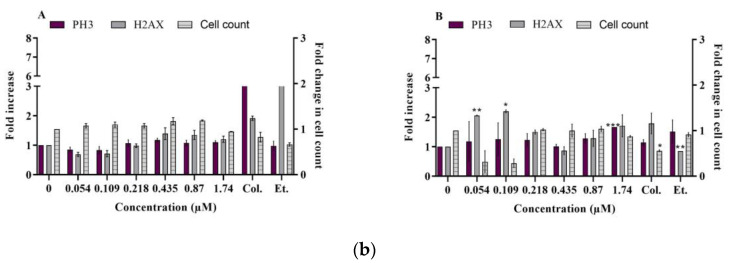
(**a**) γH2AX and phospho-H3 levels in proliferative HepaRG cells exposed to C17-SAMT for 24 h. (**A**) Experiment 1 and (**B**) experiment 2. All results are expressed as fold change compared to the negative controls. Two independent experiments are presented. * *p* < 0.05; *** *p* < 0.001; **** *p* < 0.0001. (**b**) γH2AX and phospho-H3 levels in differentiated HepaRG cells exposed to C17-SAMT for 48 h. (**A**) Experiment 1 and (**B**) experiment 2. All results are expressed as fold change compared to the negative controls. Two independent experiments are presented. * *p* < 0.05; ** *p* < 0.01; *** *p* < 0.001.

We observed a concentration-dependent increase in both γH2AX and pH3 markers in proliferating HepaRG cells following a 24 h exposure to C17-SAMT, and most remarkably at C17-SAMT concentrations of 0.87 µM and 1.74 µM (Figure 2a). In both experiments, pH3 levels increased significantly only at the highest concentration (1.74 µM). Positive controls, 1 µM etoposide, and 30 ng/mL colchicine increased levels of γH2AX and pH3, respectively. No significant decrease in cell count was observed following treatment with C17-SAMT. These results are in accordance with our findings in the MTT test (Figure S1).

Following a 48 h treatment in differentiated HepaRG cells, C17-SAMT did not induce γH2AX or pH3 (Figure 2b). As expected, etoposide induced more than a 3-fold increase in γH2AX (experiment 1, Figure 2b). Colchicine induced a 2- to −3-fold increase in pH3 (Figure 2b). All results are expressed as fold change compared to the negative controls. Two independent experiments are presented in each case (proliferating and differentiating HepaRG cells).

- Ataxia telangiectasia mutated (phospho ATM S1981)

**Figure 3:**
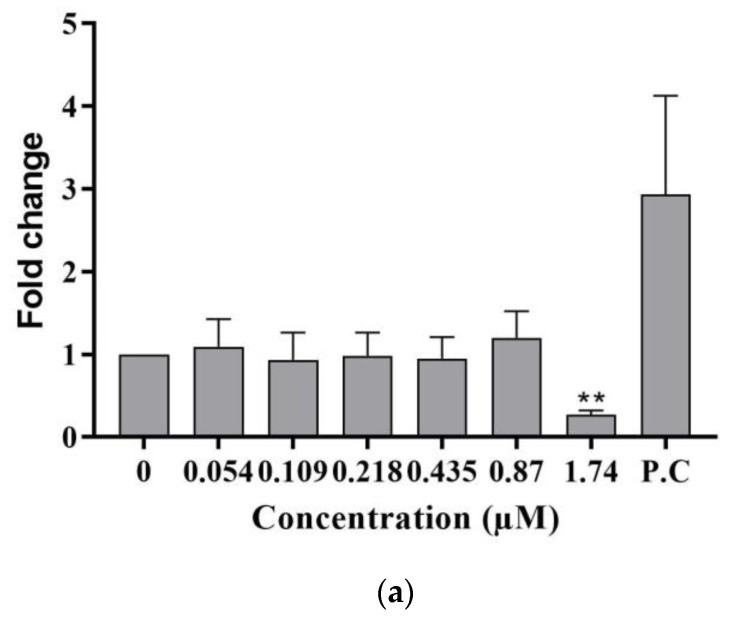

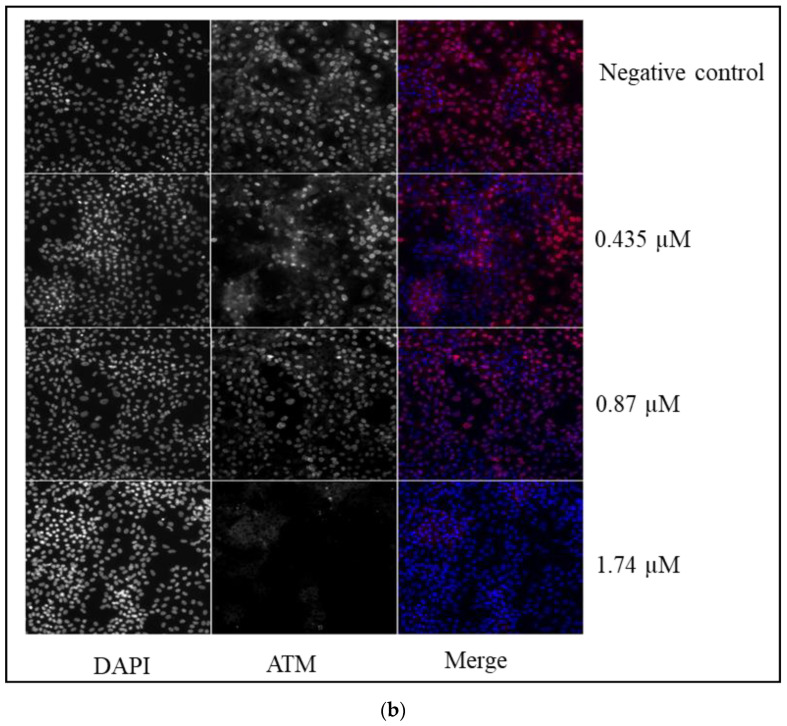
(**a**) Phospho ATM S1981 levels in differentiated HepaRG cells following a 24 h treatment with C17-SAMT. Amiodarone is used as a positive control at 100 µM. The presented results are the combination of three independent experiments with three technical replicates. ** *p* < 0.01. (**b**) Representative images representing phospho ATM immunostaining in differentiated HepaRG cells exposed to C17-SAMT for 24 h.

The DNA damage response in differentiated HepaRG cells following a 24 h treatment with C17-SAMT was also evaluated with the quantification of levels of phospho ATM S1981 (Figure 3a,b). Interestingly, a statistically significant decrease in phospho ATM S1981 was observed at 1.74 µM. The positive control, amiodarone at 100 µM, increased the levels of phospho ATM S1981 (2.93 ± 1.19) (Figure 3a). The experiment was performed three times, each with three technical replicates.

#### 2.2.2. Mitochondrial Membrane Potential (ΔΨm)

- TMRE labeling

**Figure 4:**
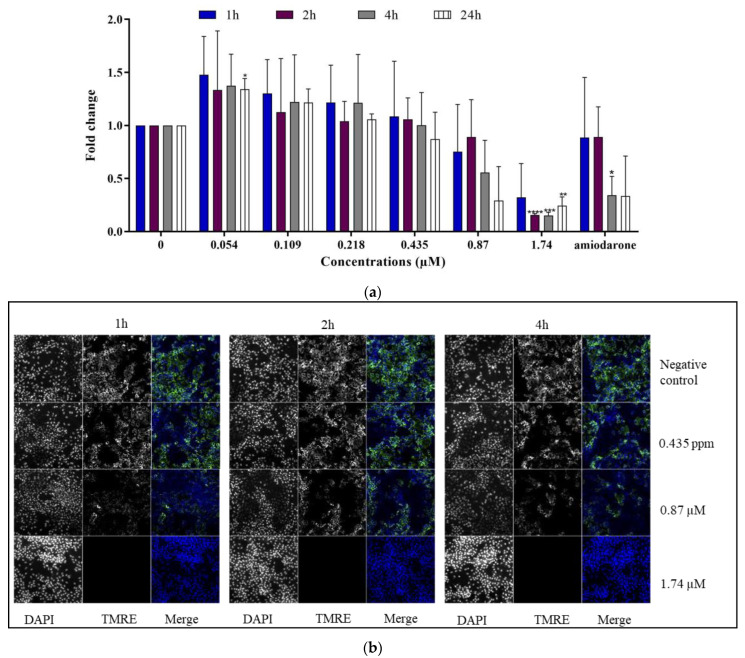
(**a**) Mitochondrial transmembrane potential in differentiated HepaRG cells after 1, 2, 4, and 24 h treatments with C17-SAMT. Amiodarone (100 µM) was used as a positive control. * *p* < 0.05; ** *p* < 0.01; *** *p* < 0.001; **** *p* < 0.0001. (**b**) Representative images of TMRE intensity in differentiated HepaRG cells following different exposure times to C17-SAMT.

A significant decrease in mitochondrial membrane potential was observed in differentiated HepaRG cells treated with 1.74 µM C17-SAMT (Figure 4a,b). The rapid decrease in ΔΨm was apparent after 1 h of treatment and became statistically significant following 2 h of treatment. The positive control, amiodarone, induced a statistically significant decrease in TMRE labeling after a 4 h exposure (* *p* < 0.05) compared to the untreated cells.

- ATP levels

**Figure 5:**
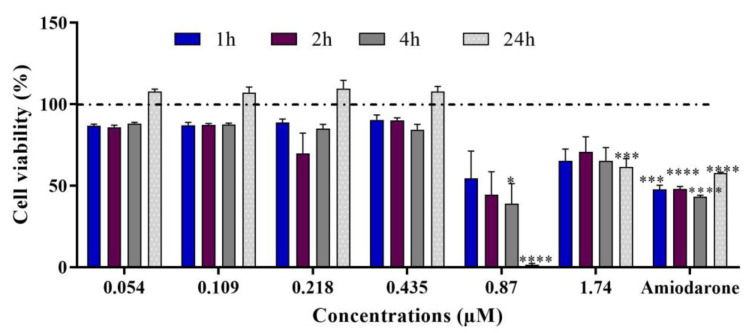
Relative ATP levels in differentiated HepaRG cells after exposure to C17-SAMT from 1 h to 24 h. Amiodarone 100 µM was used as a positive control. * *p* < 0.5, *** *p* < 0.001, **** *p* < 0.0001.

Considering the considerable decrease in ΔΨm, we then investigated the effect of the toxin on cellular ATP levels in differentiated HepaRG cells treated with C17-SAMT from 1 h to 24 h. Relative ATP levels were evaluated using the CellTiter-Glo^®^ (Promega, Wisconsin, USA) luminescent cell viability assay kit. A decrease in ATP levels was observed at 0.87 µM and 1.74 µM (Figure 5). Decreases were observed following a 1 h and 2 h exposure and were statistically significant after 4 h and 24 h. A significant decrease in ATP levels was observed at all time points in cells treated with amiodarone.

#### 2.2.3. Inflammatory Response

- NF-κB translocation

**Figure 6:**
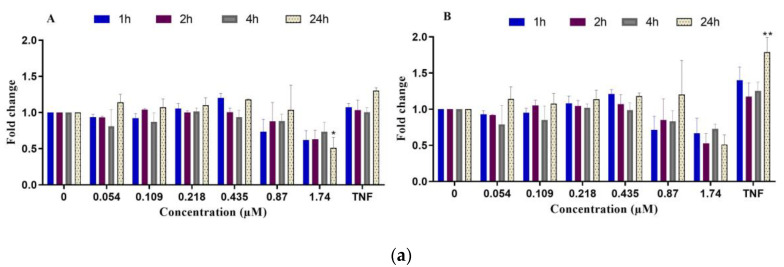

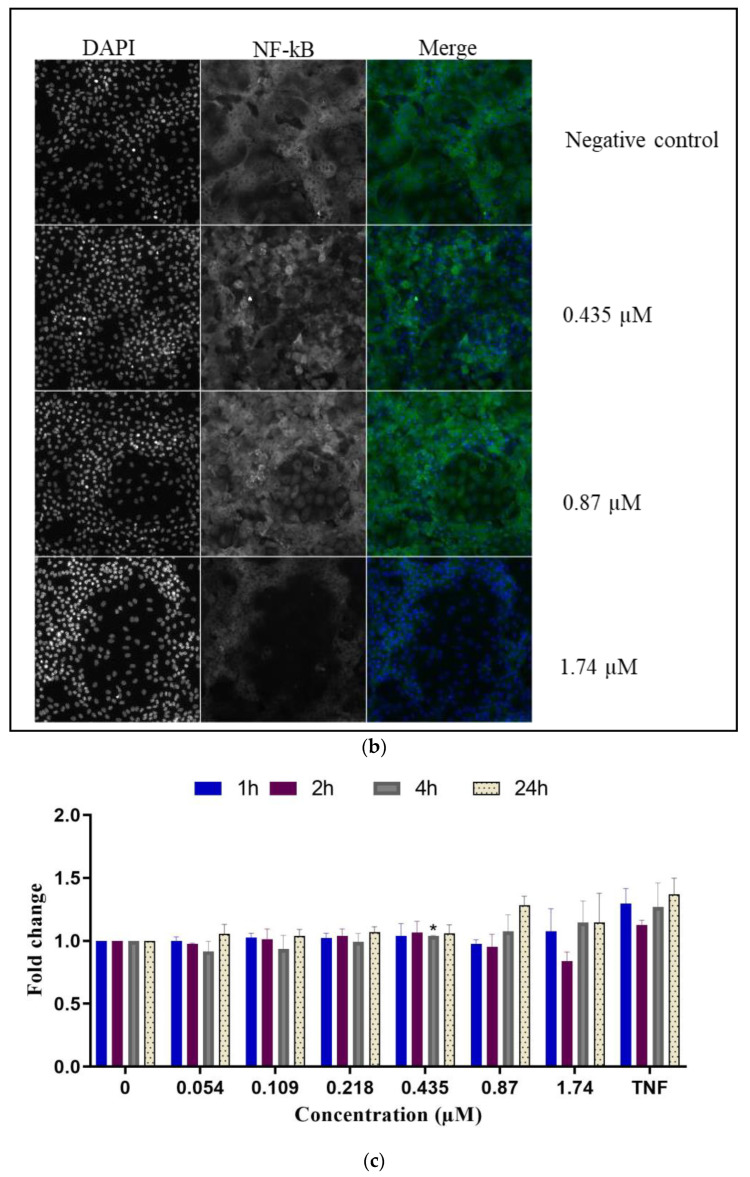
(**a**) NF-κB fold change in the cytoplasm (**A**) and nuclei (**B**) of HepaRG cells after exposure to C17-SAMT for 1 to 24 h. * *p* < 0.05; ** *p* < 0.01. (**b**) Representative images of NF-κB immunostaining intensity in HepaRG cells after 24 h of exposure to C17-SAMT. (**c**) NF-κB translocation (nucleus/cytoplasm ratio) in differentiated HepaRG cells following treatment with C17-SAMT for 1 h to 24 h. (*) *p* < 0.05.

Interestingly, significant decreases in NF-κB levels were observed at all time points following treatment of HepaRG cells with C17-SAMT, both in the cytoplasm and in the nuclei (Figure 6a,b), suggesting an overall reduction in total cellular NF-κB levels. Figure 6b illustrates the effect of the toxin on the total levels of NF-κB in HepaRG cells, with a drastic decrease in immunostaining intensity at 1.74 µM.

The nucleus/cytoplasm ratio of NF-κB labeling shows a slight increase in fold change at 0.87 µM and 1.74 µM after 24 h (Figure 6c), although this was not statistically significant. However, significant translocation was observed at 0.435 µM after 4 h of exposure to the toxin (* *p* < 0.05).

- IL-8 levels

**Figure 7:**
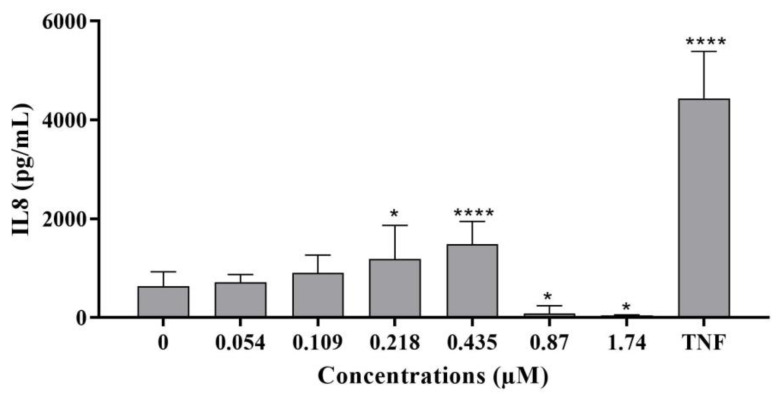
IL-8 secretion in differentiated HepaRG cells exposed for 24 h to C17-SAMT. TNF α (100 ng/mL) was used as a positive control. Mean concentrations ± SD are shown. * *p* < 0.05; **** *p* < 0.0001).

IL-8 levels were measured by an enzyme-linked immunosorbent assay (ELISA). Treatment of differentiated HepaRG cells with 0.218 and 0.435 μM C17-SAMT induced significant increases in IL-8 secretion (Figure 7) compared to untreated cells. In parallel with the decrease in the NF-κB levels in both cytoplasm and nuclei (Figure 6), IL-8 levels significantly decreased at 0.87 µM and 1.74 µM of the toxin (*p* < 0.0001 and *p* < 0.001, respectively) compared to the negative control. The positive control TNFα (100 ng/mL) induced a significant increase in IL-8 compared to the negative control.

#### 2.2.4. Oxidative Stress

- Superoxide dismutase (SOD2)

**Figure 8:**
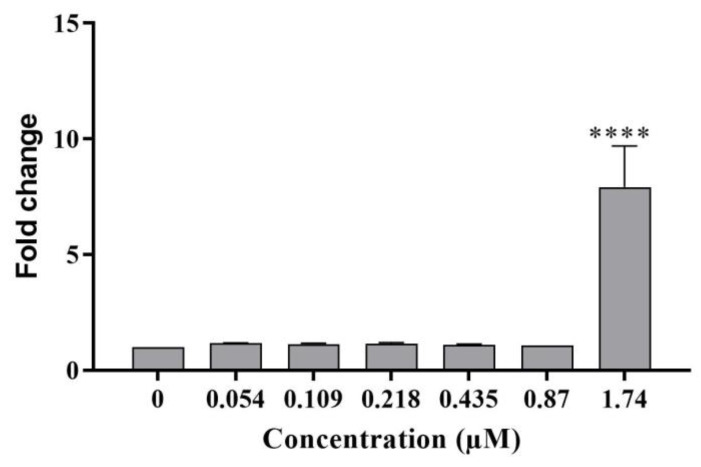

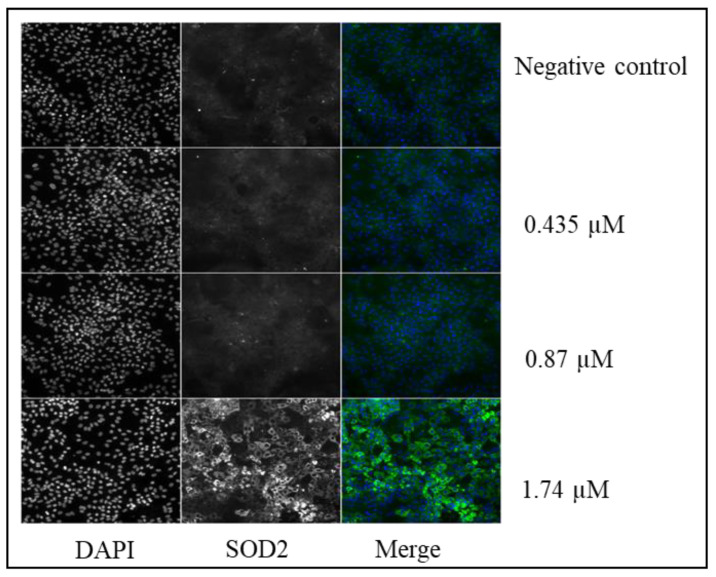
Mitochondrial superoxide dismutase SOD2 in differentiated HepaRG cells after 24 h exposure to C17-SAMT, with representative images of SOD2 immunostaining in HepaRG cells after 24 h exposure to C17-SAMT. **** *p* < 0.0001.

A statistically significant 8-fold increase in SOD2 levels was observed following a 24 h treatment of differentiated HepaRG cells with 1.74 µM C17-SAMT (Figure 8).

- Heme oxygenase-1

**Figure 9:**
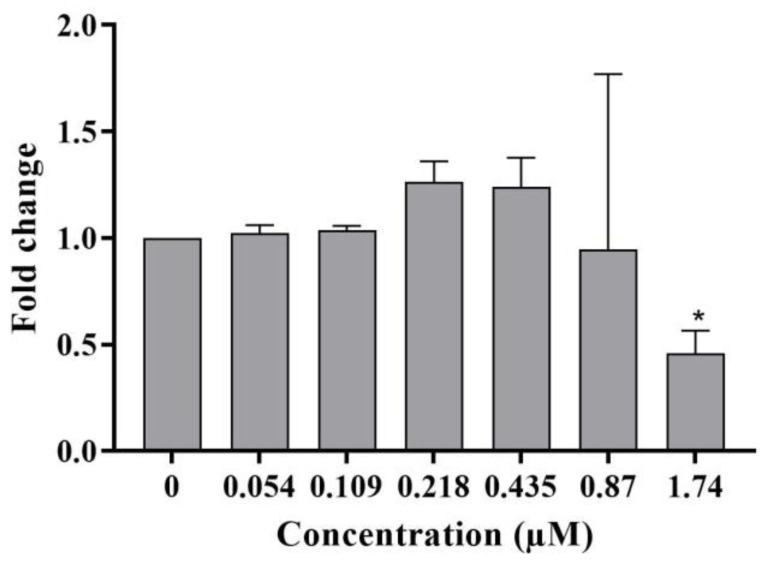
Heme oxygenase-1 levels in differentiated HepaRG cells exposed to C17-SAMT for 24 h. * *p* < 0.05.

Treatment of differentiated HepaRG cells for 24 h with C17-SAMT induced a slight increase in HO-1 expression at 0.218 µM and 0.435 µM, although this increase was not statistically significant (Figure 9). Interestingly, we observed a significant decrease in HO-1 levels at 1.74 µM.

## 3. Discussion

The aim of this study was to investigate the in vitro cytotoxic and genotoxic potential of C17-SAMT in human cell lines. A battery of in vitro tests, including the micronucleus assay in the human lymphoblastoid TK6 cell line and multiparametric cytotoxicity assays in the human hepatic HepaRG cell line, was carried out to elucidate the genotoxic and cytotoxic mechanisms of C17-SAMT.

The genotoxic potential of C17-SAMT was investigated using the micronucleus assay in TK6 cells (OECD guideline No. 487 [19]) and the quantification of γH2AX, pH3 S10, and phospho ATM S1981 immunostaining in HepaRG cells.

After a 24 h exposure to C17-SAMT, MN frequency significantly increased in TK6 cells at concentrations starting from 0.218 µM. If cell population doubling (PD) is more than 2.0 cell cycles by the end of the treatment period, RPD values tend to decrease compared to RICC values due to log transformation, therefore underestimating cytotoxicity [23,24]. In our study, as RPD values for negative controls varied between 2.2 and 2.3 for the 24 h treatment, RICC would be the best parameter for cytotoxicity estimation. In OECD guideline No. 487, genotoxicity reported at concentrations inducing cytotoxicity higher than 55 ± 5% could be related to collateral events. Therefore, higher concentrations (0.87 and 1.74 µM) of C17-SAMT associated with high cytotoxicity (above the 60% threshold) were not taken into account for mutagenicity interpretation. In our study, MN induction in TK6 cells was observed for at least two consecutive concentrations of C17-SAMT without reaching the cytotoxicity threshold and, therefore, supporting the genotoxicity of C17-SAMT. A similar conclusion was obtained on mutagenicity with the sphinganine analog mycotoxin fumonisin FB1 in rabbit kidney RK13 cells [25], HepG2 cells [26], and human peripheral blood cells [27].

Aneugenic agents mainly target non-DNA components, which can lead to dysfunction or reduced functionality during cell division. Therefore, chromosome segregation may be impaired, resulting in improper separation [28,29]. In contrast, clastogenic compounds cause breaks in DNA, and the resulting damage is detected through the biomarker γH2AX, which is produced by the phosphorylation of histone H2AX. While γH2AX phosphorylation is a marker of DNA double-strand breaks [30], studies have shown that aneugenic compounds cause phosphorylation of histone H3 that shows chromatin condensation during mitosis [31,32,33]. In our study, C17-SAMT did not induce DNA double-strand breaks in differentiated HepaRG cells following a 48 h exposure to the toxin. In contrast, in proliferative HepaRG cells, a slight increase in levels of γH2AX and phosphoH3 S10 was observed, without any decrease in cell viability. Concerning the genotoxic mode of action of C17-SAMT, our results support the aneugenic properties of the compound without ruling out clastogenicity, as described in various publications using γH2AX and phosphoH3 biomarkers [29,34]. The significant decrease in phospho ATM S1981 levels in differentiated HepaRG cells following treatment with C17-SAMT was unexpected. Typically, a decrease in phospho ATM S1981 levels is not observed, making it a very rare occurrence. Interestingly, it was shown that ATM plays a key role in DNA damage response initiation since the amplification of ATM signaling has been hypothesized to occur via a cyclic process, wherein ATM initiates the phosphorylation of H2AX, which subsequently recruits MDC1, leading to further stabilization of ATM at the chromatin region adjacent to the double-strand break (DSB). This results in the expansion of H2AX phosphorylation over megabases of DNA that flank the DSB [35], and ATM-deficient cells have been shown to exhibit disrupted mitochondrial function and reduced ATP production [36], which correlates with the findings of this study.

We observed evidence of DNA damage in proliferating TK6 cells and proliferating HepaRG cells, but not differentiated HepaRG cells. *Alternaria* mycotoxins (alternariol AOH and alternariol methyl ether AME), other analogs of the sphinganine group, induced DNA double-strand breaks when tested on liver HepG2 cells [37], proliferative human colon adenocarcinoma cell line HT29, and A431vulva carcinoma cells [38], as well as on murine RAW264.7 macrophages [39]. This suggests that the genotoxic effects of sphinganine analogs are induced primarily in proliferating cells.

C17-SAMT induced a decrease in the levels of NF-κB in the cytoplasm of HepaRG cells after a 24 h exposure. The transcription factor NF-kB is mainly found in the cytosol as an inactive complex with a subclass of inhibitory proteins known as inhibitors of NF-kB (IkB) [40,41,42]. Following a pro-inflammatory stimulus, IkB proteins are quickly phosphorylated and degraded through the proteasomal pathway. The free NF-κB subsequently translocates to the nucleus, where it interacts with kB-binding sites in the promoter regions of target genes to activate transcription [42,43]. In our study, in addition to a significant decrease in cytoplasmic NF-κB levels, C17-SAMT treatment resulted in significant decreases in the levels of NF-κB detected in the nucleus. The cause of this decrease in total cellular levels of NF-κB is not clear; however, we suggest that the toxin induces protein degradation. Recently, it was shown that NF-κB degradation can be triggered by interactions with *Pneumococcus* [44]. In fact, disruption of NF-κB signaling could be due to multiple factors, including the inhibition of subunit phosphorylation, a necessary step for NF-κB translocation [45], or through proteasomal degradation caused by the COMMD1 and 2 proteins (following a possible interaction with the toxin or its metabolites) [44,46,47]. The inflammatory response was also evaluated by measuring IL-8 cytokine secretion after a 24 h exposure to the toxin. Our results showed that C17-SAMT induced a statistically significant increase in IL-8 secretion at concentrations of 0.218 and 0.435 µM, followed by a nearly complete suppression. The decrease in total cellular NF-κB levels could, therefore, affect the transcription of cytokines responsible for the pro-inflammatory response. These results are similar to those found in *Alternaria* toxins, a family of sphinganine analogs, following treatment in the non-cancerous colon epithelial cell line HCEC-1CT [48]. It was shown that these toxins suppress the elevation of cytokine mRNA level relative to the inflammatory response and, therefore, the IL-8 secretion in a concentration-dependent manner starting at 1 µM AOH [48]. The shift between immunomodulatory and immunosuppressive potential is a characteristic of some mycotoxins depending on the tested concentration, the exposure time, and the cell type [49]. Aflatoxin B1 (AFB1), OTA, DON, T-2 toxin (T-2), FB1, and ZEA at low doses can induce an inflammatory response in vivo, but high concentrations can induce immunosuppression. Extended exposure in contrast to short-term mycotoxin exposure is immunosuppressive as well [49].

In addition, this work aimed to elucidate cellular mechanisms of toxicity of the toxin in differentiated HepaRG cells. Key parameters investigated in this study were mitochondrial dysfunction and oxidative stress. We investigated the effects of C17-SAMT on mitochondrial ATP synthesis, which involves oxidative phosphorylation requiring a transfer of protons across the inner membrane of the mitochondrion leading to a net negative charge known as the mitochondrial transmembrane potential (ΔΨm) [50]. Electron leakage is a collateral event to maintain the level of ΔΨm at around −180 mV, generating in consequence ROS. In this work, our results showed a decrease in ΔΨm labeling in HepaRG cells after exposure to increasing concentrations of C17-SAMT for 1, 2, 4, and 24 h. Disrupted mitochondrial activity is associated with a reduction in ATP levels after 4 h and 24 h and an elevation of mitochondrial stress markers. At the same time, levels of the mitochondrial antioxidant SOD2 enzyme significantly increased while HO-1 significantly decreased. Altogether, C17-SAMT leads to a disruption in mitochondrial membrane potential associated with a decrease in ATP production. This may lead to an over-production of ROS resulting in oxidative stress. This process induced a dramatic increase in the expression of SOD2 in differentiated HepaRG cells. In addition to its role in heme degradation, HO-1 is known to be a stress-response protein and is induced by several oxidative factors such as heavy metals, endotoxin, heat shock, inflammatory cytokines, and prostaglandins [51]. As well, HO-1 deficiency has been associated with fatal diseases, tissue injuries, and oxidative stress [52]. Damage associated with HO-1 deficiency was observed in specific organs or cell types, including the kidney and liver, since these organs are exposed to multiple stressors regarding their role in detoxification [52]. Our results suggest that the mitochondrion is the primary target of this toxin.

The effect on the mitochondrial membrane potential has been documented for different types of mycotoxins including FB1, which was found to inhibit mitochondrial complex I in rat primary astrocytes and human neuroblastoma (SH-SY5Y) cells at 0.5, 5, and 50 µM, resulting in a decreased mitochondrial and cellular respiration and a depolarization of the mitochondrial membrane [53]. In human hepatic HepG2 cells, a 24 h exposure to 200 µM of FB1 generated oxidative stress, including elevated ROS and SOD2 levels and depolarization of the mitochondria [54]. Fumonisin b2 (FB2), a structural analog of FB1, reduced ATP production, increased the mitochondrial stress marker HSP60, and suppressed upregulation of mitochondrial stress response proteins SIRT3 and LONP1 in human embryonic kidney HEK293 cells at 5–500 µM. Furthermore, alternariol (AOH), which is another sphinganine analog mycotoxin, was found to be responsible for strong oxidative stress by generating ROS, causing lipid peroxidation, and increasing SOD activity at 3.125 to 100 μM after a 24, 48, and 72 h treatment in human colon adenocarcinoma (Caco-2) cells [55].

The current study shows clear evidence that C17-SAMT impairs mitochondrial function, which was associated with an increase in SOD2 expression. This toxin also had significant effects on the pro-inflammatory response in differentiated HepaRG cells. In proliferating cells, a slight induction of DNA double-strand breaks was observed, as well as an induction of MN formation in TK6 cells, with a strong cytotoxic effect at the highest concentration. In a previous study [18], we have shown that C17-SAMT induced primary DNA damage in the liver of mice treated at a dose of 300 µg/kg b.w/day (three oral administrations in 45 h), which was associated with an elevated number of hepatocytes in mitosis and was an indicator of a regeneration process. The results from both the in vivo and the current in vitro studies are strongly correlated, and further investigation is clearly necessary in order to elucidate the precise molecular mechanisms associated with the toxicity of C17-SAMT; in particular, the effects of this toxin on mitochondrial function.

## 4. Materials and Methods

### 4.1. Chemicals and Reagents

#### 4.1.1. Shellfish Sampling

Farming areas in the Bizerte Lagoon are controlled by the “Commissariat Régional au Développement Agricole de Bizerte” (CRDA, Bizerte, Tunisia). Samples of mussels (*Mytilus galloprovinacialis*) were collected over several months from different farming areas in the Bizerte Lagoon. Samples were kept at 4 °C until analyzed.

#### 4.1.2. Chemicals and Reagents

C17-SAMT was purified from the contaminated mussel extract (*M*. *galloprovincialis*) using a bio-guided approach. As described previously by Marrouchi et al. [17], HPLC purification coupled with mouse bioassays was carried out to obtain the purified toxin. Briefly, an Agilent 1100 series analyzer with a Hypersil ODS-2 column (C18, 4.6 m, 250 mm, 5 m, ThermoScientific, Illkirch, France) was used to calculate the toxin concentration. D-erythrosphinganine (C17-SPA) from Avanti Polar Lipids (Alabaster, AL, USA) and a certified C17-SPA (10 mg/mL) solution were used to calibrate, and peak areas were measured to calculate peak intensities. The purified fraction of the toxin was kept at −20 °C until it was analyzed.

Primary and secondary antibodies for HCA experiments were provided by Abcam (Cambridge, UK). Primary antibodies included rabbit polyclonal anti-histone H3 phospho S10 (ab5176), mouse monoclonal anti-γH2AX (ab26350), mouse monoclonal anti-phospho ATM S1981 (ab19304), rabbit polyclonal anti-NFκB (ab16502), and rabbit polyclonal anti-HO1 (ab13243). Secondary antibodies used were goat anti-rabbit IgG H&L, Alexa Fluor^®^ 488 (ab96891) and goat anti-mouse IgG H&L, Alexa Fluor^®^ 647 (ab96876). A rabbit polyclonal anti-SOD2 antibody (PA5-30604) was provided by Invitrogen (Invitrogen™, Illkirch, France).

#### 4.1.3. Cell Culture

- TK6 cells

The human TK6 lymphoblastoid cell line (ECACC, lot n°17E062) was maintained as a suspension culture in Glutamax™ RPMI 1640 medium supplemented with 10% fetal calf serum (FCS), 100 IU/mL penicillin, and 100 µg/mL streptomycin.

- HepaRG cells

HepaRG cells were cultured, as previously described [56], with slight modifications. Cells were grown in William’s E medium supplemented with 10% fetal calf serum (FCS), 100 U/mL penicillin, 100 μg/mL streptomycin, 2 mM glutamine, 5 μg/mL insulin, and 50 μM hydrocortisone hemisuccinate. HepaRG cells (passages 13–19) were seeded at a density of 26,000 cells/cm^2^ in 96-well plates.

Non-differentiated HepaRG cells were treated 24 h after plating.

Differentiated HepaRG cells were obtained after 4 weeks of culture (2 weeks with complete medium alone followed by 2 weeks with the addition of 1.7% DMSO).

#### 4.1.4. Micronucleus Assay in TK6 Cells

The micronucleus assay was performed following the recommendations of OECD guideline No. 487 [19], as previously described [57]. TK6 cells (2 × 10^5^ cells/mL, 12-well plates) were exposed to C17-SAMT for 24 h in a 5% FCS culture medium. Following the 24 h treatment, cells were collected with no recovery period. Methyl methane sulfonate (MMS) (Sigma-Aldrich, St. Quentin-Fallavier, France) at 10 µg/mL was used as a positive control. Cells were collected by centrifugation at 136× *g* for 5 min and counted using Trypan blue exclusion for cytotoxicity examination. The relative increase in cell count (RICC) and relative population doubling (RPD) were calculated according to OECD Guideline No. 487. Before fixation with an ethanol acetic acid solution (ratio 3:1 (*v*/*v*)) for 10 min at room temperature, cells were submitted to a hypotonic shock for 4 min using an RPMI medium and distilled water (ratio 1:1 (*v*/*v*)). Cells were centrifuged again, resuspended in the fixative solution, spread on glass slides, and stained with acridine orange (67 µg/mL in PBS). Finally, micronuclei were scored in 2000 mononucleated cells per experiment (2 independent experiments in duplicates) under a fluorescence microscope (Leica DMR, Wetzlar, Germany).

#### 4.1.5. High Content Analysis

Following treatment of HepaRG cells, cells were fixed with 4% formaldehyde for 15 min at room temperature. Plates were then washed twice with PBS and permeabilized with PBS 0.2% Triton X-100 for 10 min at room temperature. Cells were then incubated for 30 min in a blocking solution (PBS 1% BSA and 0.05% Tween-20) before the addition of primary antibodies. Primary antibodies, anti-PH3, and anti-γH2AX were diluted 1:2000 in a blocking solution. The anti-ATM and anti-NFκB antibodies were diluted at 1:1000, and the rabbit polyclonal anti-HO1 and anti-SOD2 antibodies were diluted at 1:500. Plates were incubated with primary antibodies overnight at 4 °C and then washed twice with PBS/0.05% Tween-20. Plates were incubated with secondary antibodies diluted at 1:2000 in PBS/1% BSA/0.05% Tween-20 for 1 h at room temperature. Nuclei were stained with DAPI (1 µg/mL in PBS/0.05% Tween-20) (Sigma-Aldrich, Saint-Louis, MO, USA, D9542) for automated cell identification using an Arrayscan VTi (ThermoScientific, Waltham, MA, USA). Plates were then washed with PBS, and 100 µL PBS was added to each well.

Images from eight fields (20× magnification) per well were analyzed for quantification of fluorescence at 650 nm and 488 nm (three independent experiments in duplicates).

Immunofluorescence of γH2AX, H3 phospho S10, and phospho ATM S1981 markers were quantified in the nucleus using the Target Activation module of the BioApplication software (ThermoScientific, Waltham, MA, USA). SOD2 and HO-1 levels were quantified in the cytoplasm of HepaRG cells, and nuclear and cytoplasmic levels of NF-κB were quantified using the Compartmental Analysis Bioapplication. Data were expressed as fold changes with respect to the negative control for scoring.

#### 4.1.6. Mitochondrial Activity

- Mitochondrial transmembrane potential: TMRE

After exposure to purified C17-SAMT, differentiated HepaRG cells were incubated for 30 min with 50 nM TMRE (Sigma-Aldrich, Saint-Louis, MO, USA, #87917) and 3 µg/mL Hoechst (Invitrogen™, Illkirch, France, #43570). Before screening the plates, cells were washed with PBS, and a serum-free medium was added to each well immediately prior to quantification.

- ATP levels

A CellTiter-Glo^®^ (Promega, WI, USA) luminescent cell viability assay kit was used to determine the levels of ATP in HepaRG cells after toxin exposure. Following treatment, the plate was left at room temperature for 30 min, and 100 µL of a CellTiter-Glo reagent was added to each well. Following a 10 min incubation with agitation, luminescence was measured with a Fluostar Optima^®^ plate reader (BMG Labtech, Elancourt France). Relative ATP levels were then calculated compared to the negative control.

Relative ATP Level [%] = [Luminescence [sample]/Luminescence [control]] × 100

#### 4.1.7. IL-8 Enzyme-Linked Immunosorbent Assay (ELISA)

The secretion of IL-8 in differentiated HepaRG cells following exposure to C17-SAMT for 24 h was measured by ELISA.

Briefly, plates (Maxisorp NUNC44240) were coated with the capture antibodies (AC IR Human IL8 Mab, M801, ThermoFisher, Grigny, France) at 4 °C overnight and then washed 3 times with 100 μL/well PBS-0.05% Tween-20 buffer. Coated plates were blocked with 100 μL of a Super Block Buffer blocking solution (Thermofisher, 37515) for 1 h at room temperature. After washing three times, 90 μL of the diluted medium was added to each well and incubated at RT for 90 min with continuous agitation, followed by three washing steps. Wells were then incubated with 50 μL of the diluted secondary biotinylated antibodies (IL-8 Biotinylated, ThermoFisher, M802) for 1 h, followed by three washes with PBS-Tween. For spectrophotometric detection, wells were incubated with 100 µL of a streptavidin peroxidase solution (ThermoFisher, ref21132). Following washes, plates were incubated with 100 μL/well 3,3′,5,5′-Tetramethylbenzidine TMB (ThermoFisher, 34028) for 45 min at RT. The reaction was terminated by the addition of 50 μL/well 1 M H_2_SO_4_. The absorbance at 405 nm was quantified using a Fluostar Optima^®^ plate reader (BMG Labtech, Elancourt, France).

#### 4.1.8. Statistical Analysis

The statistical analyses in this study were conducted using GraphPad PRISM 5 software (GraphPad Software Inc., San Diego, CA, USA). Multiparametric cytotoxicity assays in HepaRG cells were analyzed for fold changes relative to negative controls using a one-way ANOVA, followed by a Dunnett post-hoc test. To compare the proportion of micronucleated cells in treated and negative control cultures, a chi-square test with Yates’ like correction was employed for TK6 cells. The data were considered significantly different at *p* < 0.05 in all tests.

## 5. Conclusions

In conclusion, our study demonstrates that C17-SAMT is capable of inducing chromosome or genome mutation by increasing the MN formation in TK6 cells. Moreover, it exerts genotoxic effects in proliferative HepaRG cells, increasing the levels of γH2AX and phospho-H3 S10 and decreasing the levels of phospho ATM S1981. Treatment of differentiated HepaRG cells with C17-SAMT induced mitochondrial dysfunction associated with increases in markers of oxidative stress and decreases in cellular ATP levels. Moreover, C17-SAMT exhibits both immunomodulatory and immunosuppressive effects at low and high concentrations, respectively. Further studies are, therefore, needed to understand the metabolic and molecular pathways involved in toxicity.

## Data Availability

Not applicable.

## Supplementary Materials

The following supporting information can be downloaded at https://www.mdpi.com/article/10.3390/ijms24097805/s1.
