# Supplementary figures and images for: An Evaluation of the Cytotoxic and Genotoxic Effects of the Marine Toxin C17-SAMT in Human TK6 and HepaRG Cell Lines
Source: Int J Mol Sci. 2023 Apr 25;24(9):7805. doi: 10.3390/ijms24097805 (PMC10177896; doi:10.3390/ijms24097805)

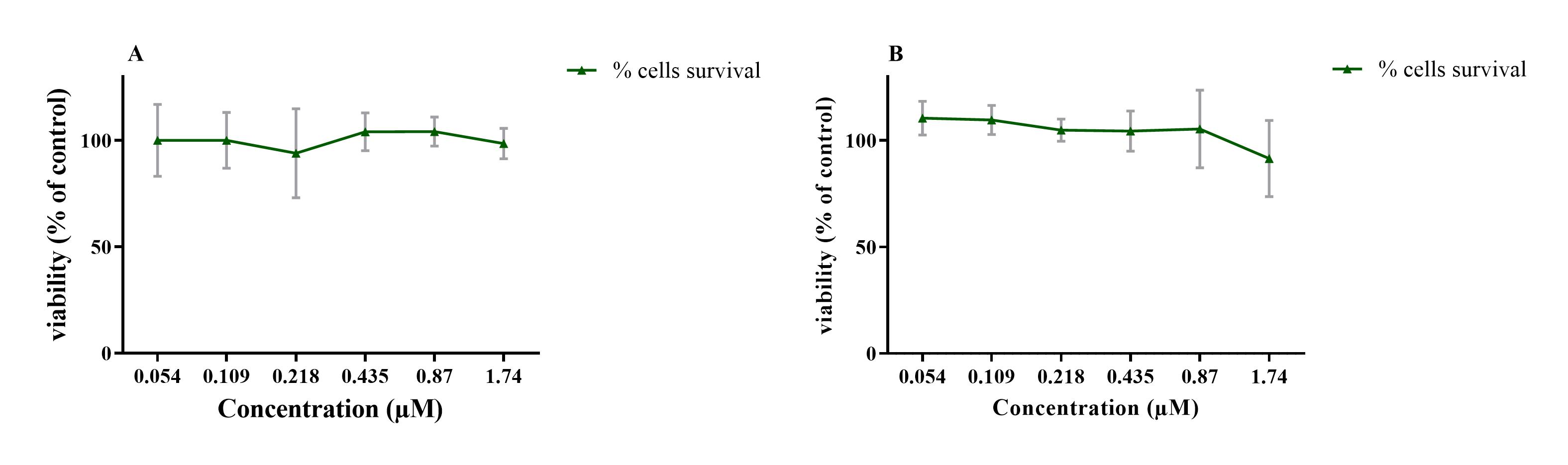

Supplement: Supplementary file 1 [file ijms-24-07805-s001.zip › Figure S1.jpg]
